# Antibiotics inhibit sphere-forming ability in suspension culture

**DOI:** 10.1186/s12935-016-0277-6

**Published:** 2016-02-12

**Authors:** Sébastien Relier, Laura Yazdani, Oualid Ayad, Armelle Choquet, Jean-François Bourgaux, Michel Prudhomme, Julie Pannequin, Françoise Macari, Alexandre David

**Affiliations:** CNRS, UMR-5203, Institut de Génomique Fonctionnelle, 34094 Montpellier, France; INSERM, U1191, 34094 Montpellier, France; Université de Montpellier, 34094 Montpellier, France; Service d’Hépato-Gastroentérologie, CHU Carémeau, Nîmes, France; Service de Chirurgie Digestive, CHU Carémeau, Nîmes, France

**Keywords:** Antibiotics, Sphere-forming ability, Suspension culture, Cancer stem cells

## Abstract

**Background:**

This last decade, a lot of emphasis has been placed on developing new cancer cell culture models, closer to in vivo condition, in order to test new drugs and therapies. In the case of colorectal cancer, the use of patient biopsies to seed 3D primary cultures and mimic tumor initiation necessitates the use of antibiotics to prevent microbial intestinal contamination. However, not only long term use of antibiotics may mask the presence of low levels of microbial contamination, it may also impact cancer cell phenotype.

**Methods:**

In this study we tested the impact of penicillin-streptomycin cocktail addition in both monolayer and suspension culture. To ensure the reliability of our observations we used six different cell lines and each experiment was performed in triplicate. Results were analyzed with Student’s t test.

**Results:**

We show that penicillin–streptomycin cocktail inhibits the sphere-forming ability of six cancer cell lines in suspension culture though it has no impact in monolayer culture. We correlate this effect with a significant decrease of cancer stem cells pool which holds self-renewal potential.

**Conclusions:**

Overall, this study warns against systematic addition of antibiotics in growth medium and raises the interesting possibility of using antibiotics to target cancer stem cells.

## Background

Despite significant advances in diagnostics and therapeutic treatment, colorectal cancer (CRC) remains a major cause of mortality worldwide [1]. This is due to the fact that CRC survival is highly dependent upon stage of disease at diagnosis: though early stage show 70–90 % 5-year survival, once the tumor spreads out to distant organs, 5-year survival plummets toward 10 % [2]. Therefore, there is a real need for a better understanding of molecular and cellular events triggering metastatic process and the conception of new, adapted, therapeutic strategies. This need led to the development of suspension culture systems, such as tumorsphere formation assay, mirroring in vivo tumor initiation and heterogeneity. The growth of microtumor-like spheroids in non-adherent culture and serum free conditions [3] necessitates the acquisition of “cancer stem cell phenotype” which lends self-renewal, multipotency, chemoresistance, and metastatic properties [4]. In the case of CRC, this cell culture model is often used as a surrogate to evaluate tumorigenic potential [5].

Antibiotics are often used in cell culture in order to prevent contamination with microbiological organism. However, this practice remains controversial since the routine use of antibiotics may favor the development of resistant strains and cryptogenic contaminants such as mycoplasmae or viruses. Penicillin–streptomycin (P/S) cocktail is the only one “recommended” by American Type Culture Collection (ATCC), though this bioresource center avoids using any antibiotic for routine cell culture and warns again long-term usage of it. While the use of this antimicrobial combination in regular culture condition is dispensable, it becomes a prerequisite when trying to establish cell line from colorectal tumor biopsies, heavily contaminated with microorganisms. The effect of P/S addition in three-dimensional (3D) cultures of cancer cells has never been addressed while several publications have raised concerns regarding the use of antibiotics, especially in serum-free conditions [6–15]. In order to improve sphere culture conditions—that favors cancer stem cell survival and growth—we tested the impact of antibiotics on suspension culture. We show that P/S addition triggers a striking decrease of sphere formation in six cancer cell lines from three distinct tissue origins. The magnitude of this effect is proportional to P/S concentration and correlates with reduced cancer stem cell population.

## Results

**Fig. 1:**
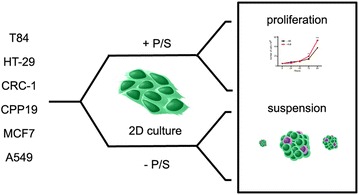
Schematic representation of the experimental protocol

**Fig. 2:**
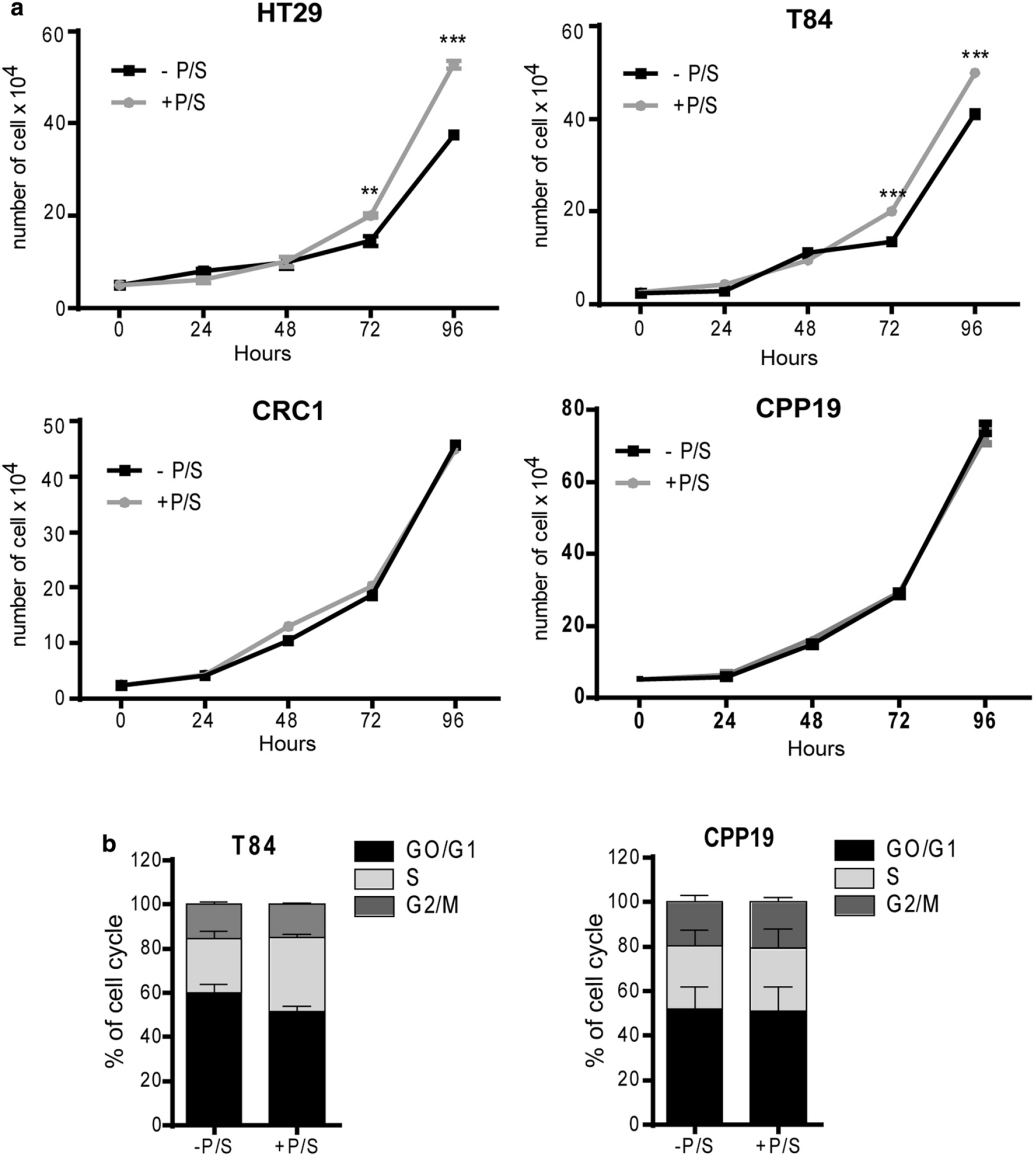
Effect of P/S on cell proliferation and cell cycle. **a** Cell proliferation was analyzed in the presence or not of antibiotics. Results are the mean ± SEM of triplicate and representative of four separate experiments. **b** Cell cycle was analyzed by flow cytometry on T84 and CPP19 cell lines. Results are the mean ± SEM of three separate experiments

**Fig. 3:**
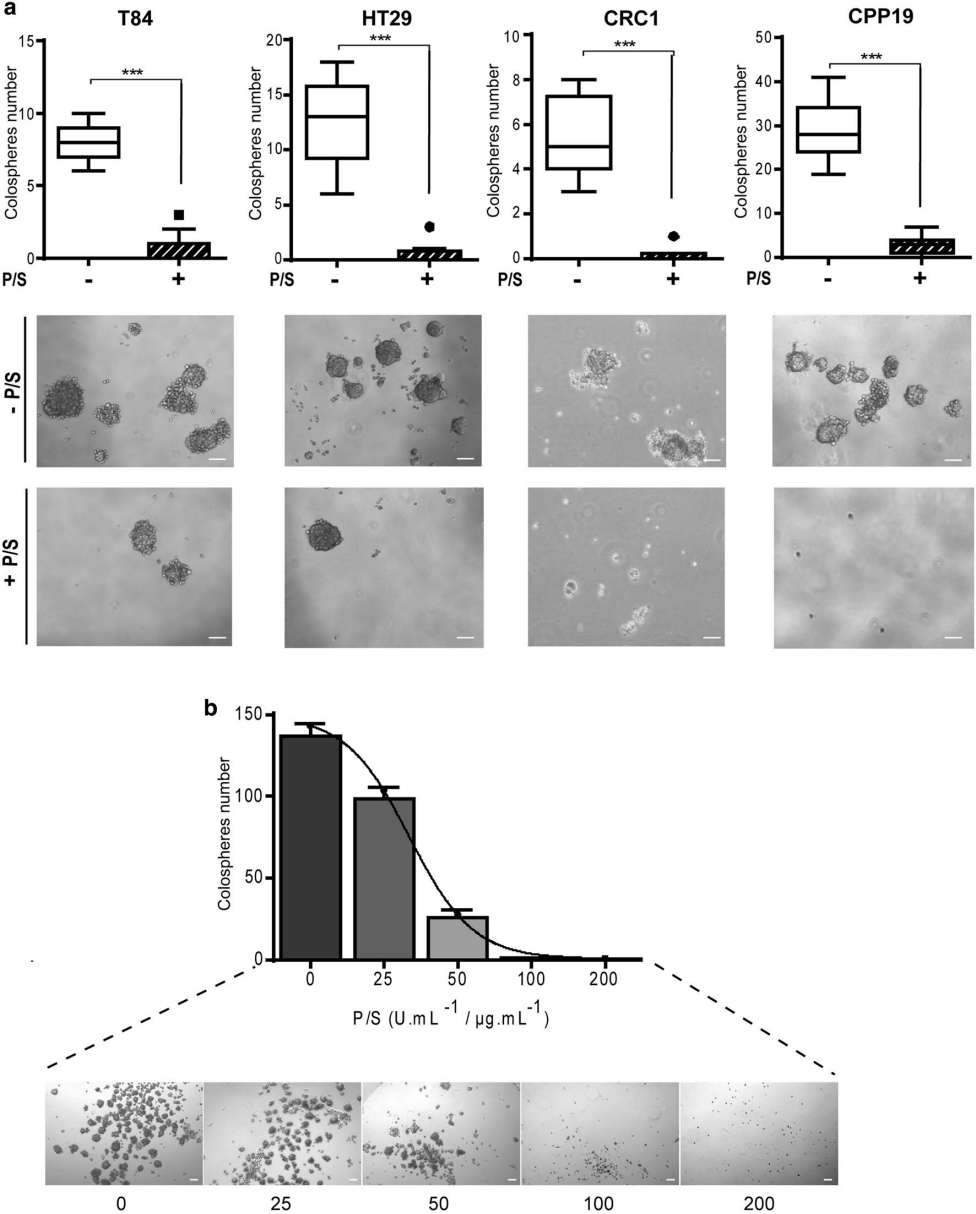
Impact of P/S addition on suspension culture. **a** T84, HT-29, CRC1 and CPP19 cell lines were passaged in suspension culture and tested for their ability to form spheres. *Tukey boxplots* that represent the first and the third quartiles by the *upper* and *lower* *horizontal lines* in a *rectangular box*, inside of which the *horizontal line* represents the median. ***p < 0.001, two tailed unpaired t test; Values are representative of three experiments. *Pictures* show representative field. *Scale bar* 100 µm. **b** Dose–response effects of P/S on *sphere* forming ability of T84 cell line in suspension culture. Results are representative of three distinct experiments

**Fig. 4:**
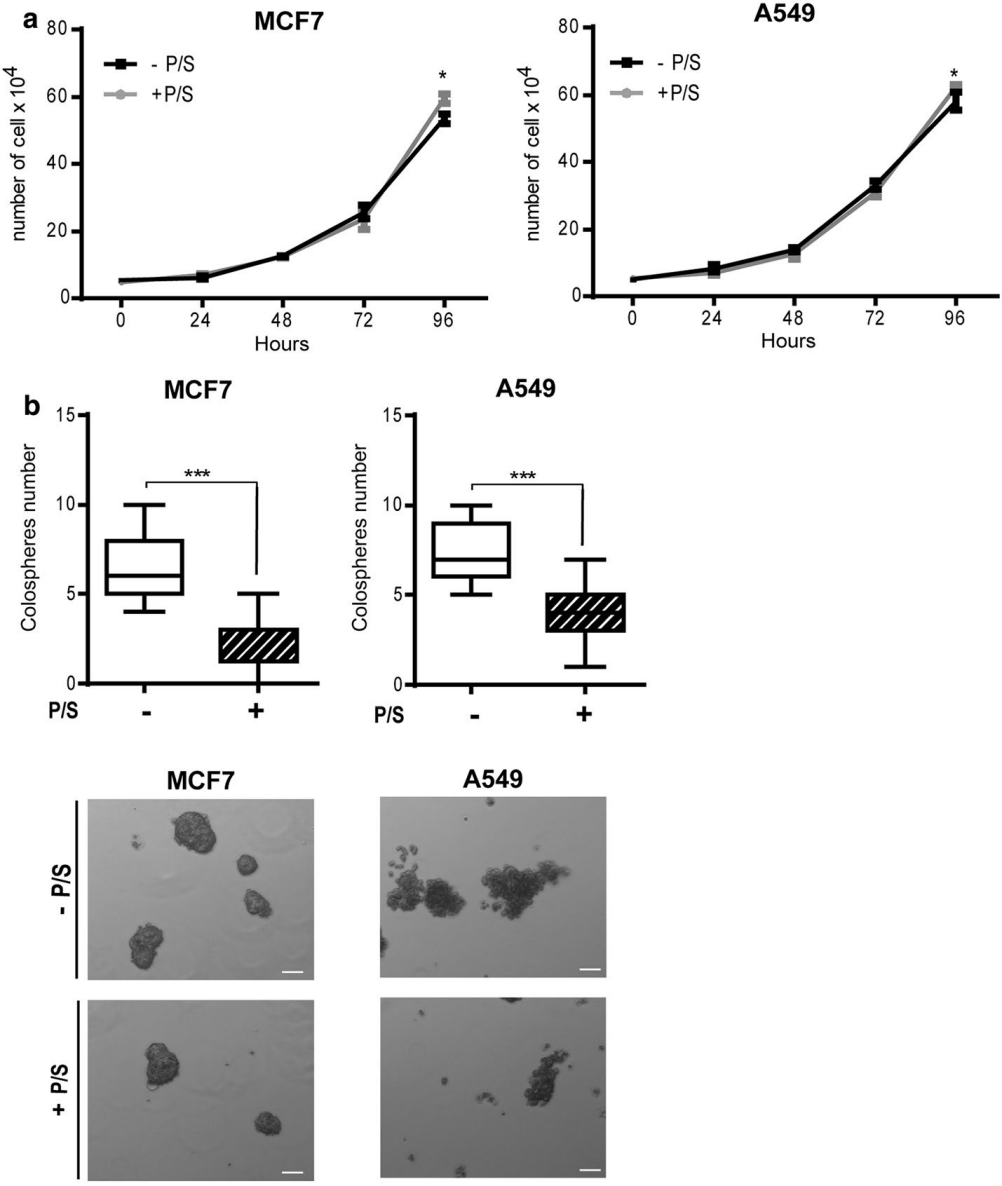
Effect of P/S on other cancer types. **a** Cell proliferation analysis of MCF7 and A549 cell lines in the presence or not of antibiotics. Results are the mean ± SEM of triplicate and representative of three separate experiments. **b** Ability of MCF7 and A549 cell lines to form *sphere* in suspension culture. *Tukey boxplots* as in Fig. 3. Values are representative of three experiments. ***p < 0.001, two-tailed unpaired t test. *Pictures* show representative field. *Scale bar* 100 µm

**Fig. 5:**
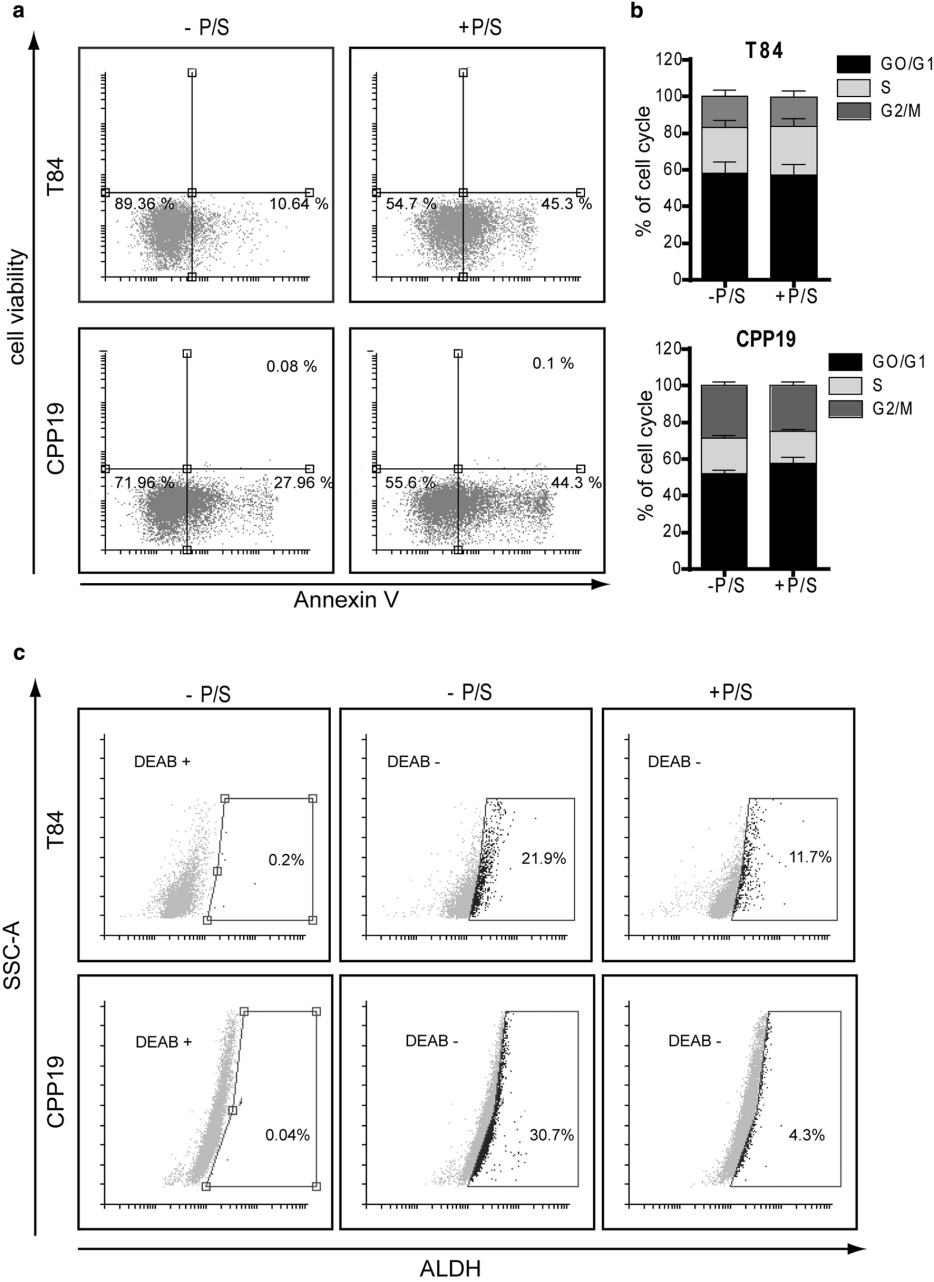
P/S addition does not affect cell cycle but targets ALDH + cells. Cell death (**a**) and cell cycle (**b**) were evaluated by flow cytometry analysis on T84 and CPP19 cell lines cultured in suspension condition. Results are the mean ± SEM of triplicate and representative of three separate experiments. **c** ALDH enzyme activity in T84 and CPP19 cell lines grown in suspension culture was analyzed by flow cytometry. As a negative control, cells were treated with the specific ALDH inhibitor DEAB. The gated cells represent the ALDH positive cells

In order to establish a broad picture of P/S effects on CRC cell growth, we chose four different cell lines: HT29, a colon adenocarcinoma grade II cell line; T84, a cell line derived from a lung metastasis of a colon carcinoma; CRC-1, a cell line derived in our lab from freshly isolated colorectal adenocarcinoma, CPP19, another cell line derived in our lab from hepatic metastasis of a CRC patient. Each of these cell lines was grown in monolayer culture for 2 weeks minimum, in the presence or absence of antibiotics, before being tested (Fig. 1). First, we tested the impact of P/S on cell growth in classical adherent conditions. Cell number was counted, in the presence or absence of antibiotics on a daily basis for 4 days (Fig. 2a). P/S slightly increased cell proliferation in HT29 and T84 cells, but had no effect in CRC-1 and CPP19 cells, underlining a subtle but noteworthy differential effect according to tumor cell origin. Accordingly, P/S addition did not impact cell cycle (Fig. 2b). Then, we tested the effect of antibiotic addition on cell suspension culture. Unlike monolayer condition, suspension culture favors the growth of tumor cells that possess sphere forming ability in serum-free medium at low cell density. Remarkably, suspension culture was severely impacted by P/S addition that triggered about fivefold decrease in sphere formation (Fig. 3a). This effect did not depend on the nature of cell lines and patient stage since the four cell lines tested were sensitive to a similar extend. Furthermore, inhibition of sphere forming ability correlates nicely with increasing concentrations of P/S and illustrates a typical dose-response relationship (Fig. 3b). In order to test whether this observation could be extended to other cancer types, we tested two other cell lines: A549, a lung epithelial carcinoma, and MCF7, a cell line derived from a pleural metastasis of breast adenocarcinoma. As expected, while P/S addition did not impact cell proliferation in monolayer culture (Fig. 4a), it triggered a significant inhibition of sphere forming efficiency in both MCF7 and A549 cell lines (Fig. 4b). In both T84 and CPP19 cells, decreased sphere forming potential in the presence of P/S correlated with elevated cell apoptosis (Fig. 5a). Depending upon the cellular context, cell cycle disturbance may trigger apoptosis [16]. However P/S addition did not seem to impact cell cycle, as shown by propidium iodide staining (Fig. 5b). This last observation—combined with the fact that antibiotics do not affect cell proliferation in monolayer culture- led us to think that P/S may not have a broad effect, but instead a selective one, impacting specific cell sub-population(s). Spheres forming ability in suspension culture is a proxy for in vivo tumorigenesis and relies on tumor initiating cells (TIC) proportion in the cell line. TIC, also called “cancer stem cells”, drive tumorigenic process through their self-renewal ability and their “plasticity” that permits to give rise to all cell types found in a particular tumor sample [17]. Though there is no ideal membrane marker for TIC, a growing number of studies have shown that high aldehyde dehydrogenase (ALDH) enzyme activity is associated with enhanced self-renewal capacity and in vivo tumorigenicity [18], both hallmarks of TIC. In T84 and CPP19 cells cultured in suspension condition, P/S addition triggered a significant decrease of ALDH positive cells (Fig. 5c) which is consistent with reduced sphere number (Fig. 3a). Therefore, P/S effect on cell suspension culture could well arise from specific impairment of self-renewal ability which is indispensable for TIC survival.

## Discussion

The goal of this study was to evaluate the impact of P/S addition on 3D culture of cancer cell lines. We focused our attention on commonly used combination of penicillin and streptomycin. By opposition with previous studies [19], P/S addition did not impact cell proliferation in regular growth condition for any of the cell lines tested. However, this study revealed a severe decrease of sphere number following P/S addition. As suggested by ALDH labeling experiments, this effect might result from a specific decrease of TIC population which nucleates sphere formation. We speculate that the loss of TIC phenotype triggers cell apoptosis, perhaps through resensitization to anoikis [20]. Altogether, this study warns against systematic addition of antibiotics in growth medium, especially in suspension culture where their usage is counter-productive and prone to artifacts.

Preventing contamination is a constant challenge in cell culture that needs to be addressed with caution. Sole reliance on antibiotics leads to poor aseptic techniques, increased mycoplasma contamination, increased antibiotic resistance among contaminants, and—as exemplified in this study—experimental bias. Therefore, what other alternative to P/S addition might there be to prevent growth medium contamination? From a practical standpoint, most general cell culture can be usually done in the absence of antibiotics, though it requires a basic culture management, tailored to meet the specific needs of the laboratory’s working conditions. Achieving and maintaining good aseptic technique is key, especially when working with valuable cultures. Nevertheless, under some circumstances—such as cell isolation of human biopsy—a decontamination procedure cannot be avoided. In any event, antibiotics should only be used as a last resort for short term applications, and they should be removed from the culture as soon as possible. Finally, we recommend caution in using antibiotics from aminoglycoside family (e.g., streptomycin, gentamycin, kanamycin) whose intrinsic RNA-binding properties may impact gene expression and cell properties. Halving the concentration of antibiotics is an acceptable possibility to consider: as shown in this study, the recommended working concentration of streptomycin ranges from 30 to 100 µg/mL which translates into significant differences in term of sphere forming ability (Fig. 3b).

From a functional perspective, we envision three possibilities in which streptomycin plays a pivotal role by associating with RNA structure(s).

**Fig. 6:**
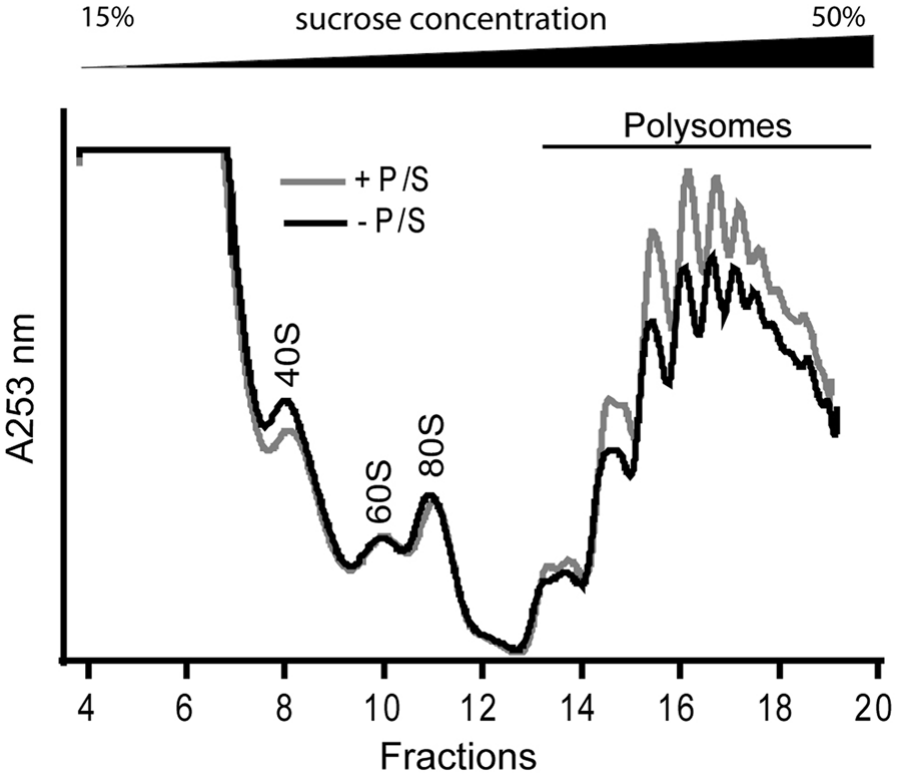
Polysome profiles from T84 monolayers grown with or without P/S. Briefly, cell lyzates were fractionated on 15–50 % sucrose gradient to isolate polysomes. Absorbance profile was continuously measured at 254 nm

First, while streptomycin specifically targets 30S prokaryotic ribosomal subunit, it can also associate with eukaryotic ribosomes when certain structural conditions are met [21–23]. By analyzing polysome profile from monolayers cultures of T84 cells, we did not observe any quantitative effect on global protein synthesis following P/S addition (Fig. 6). However, it does not discard the possibility of a subtle and “qualitative” change, which may impact a minor population of streptomycin-sensitive ribosomes.

Second, streptomycin could affect mitochondrial translation and most particularly that of TIC. This view is in line with a recent report suggesting that antibiotics targeting prokaryotic ribosome specifically impact mitochondrial activity in cancer stem cells and trigger cell death [24]. Nevertheless, it does not explain why mitochondrial activity is not impacted in monolayer culture, where high proliferation necessitates elevated protein synthesis rate, one of the most energy consuming processes in the cell.

Finally, streptomycin could associate with certain RNA hairpin loops and interfere with RNA processing/function. Indeed, while aminoglycosides are mainly used as therapeutic agents to target bacteria, they can bind in a non-selective manner to a variety of RNA that possess characteristic stem-loop structures, such as viral or non-coding RNA [25, 26]. A recent study raises the interesting possibility that aminoglycosides could bind to oncogenic miRNA precursors—that possess stem-loop structure—and inhibit their processing into fully functional miRNA [27]. This hypothesis is plausible given the complexity of cancer cell [28] and the great importance of miRNA networks in the regulation of TIC properties [20, 29].

## Conclusions

Overall, this study warns against routine usage of antibiotics whose “off-target” effects—hitherto poorly understood—may bias experimental results. While the precise mechanism by which P/S inhibits sphere-forming ability remains to be elucidated, it moots the exciting possibility of using antibiotics as tools for anti-TIC therapies. This idea is particularly attractive when considering that many antibiotics are already FDA approved.

## Methods

### Cells and ethics statement

Patient-derived cell culture of colon cancer cells (CRC-1 and CPP19) were obtained from CRC biopsies provided by CHU-Carémeau (Nîmes, France) within an approved protocol (ethical agreement no 2011-A01141-40). Signed informed consents were obtained from patients prior to samples acquisition in accordance with all ethical and legal aspects. The «Agence nationale de sécurité du médicament et des produits de santé » (ANSM) reviewed and approved this study.

Tumors were washed, minced into fragments (<2 mm^3^), digested with 0.26 U/ml of liberase H (Roche), and resuspended in Accumax (Sigma-Aldrich). Following 2 h at 37 °C, cell suspension was filtered through a 40 mm mesh and plated in DMEM medium (Invitrogen), supplemented with 10 % fetal calf serum (FCS), glutamine, antibiotics (P/S) and non-essential aminoacids. When a monolayer of patient-derived tumour cells was formed, cells were detached using trypsin/EDTA and resuspended in DMEM with 10 % FCS.

T84 and HT-29 cells were purchased from American Type Culture Collection (ATCC).

### Cell culture and cell proliferation

T84, HT-29 CRC-1, CPP19, A549 and MCF7 cell lines were cultured at 37 °C under humidified 5 % CO_2_ in DMEM medium (Invitrogen) or RPMI (Invitrogen, for MCF7 and A549) supplemented with 10 % fetal calf serum (Invitrogen), 2 mM glutamine, with or without antibiotics: 100 μg/ml streptomycin and 100 U/ml penicillin (Invitrogen). For cell proliferation analyses, 5 × 10^5^ cells were cultured in triplicate in a 12 well plate for 24, 48, 72 or 96 h. Cells were then resuspended in DMEM with 10 % FCS, after Trypsine—EDTA treatment, for counting.

### Sphere formation assays

Number of Cell Forming Sphere were determined after plating of 1000 cells/ml M11 medium (DMEM/F12 (1:1) Glutamax medium, N2 Supplement, Glucose 0.3 %, insulin 20 µg/ml, hBasic-FGF 10 ng/ml, hEGF 20 ng/ml), with or without antibiotics (Penicillin G 100 U/ml, Streptomycin 100 µg/ml) in ultra-low attachment p24-well plates (11 wells/condition) (Corning). Spheres >50 µm diameter were counted after 6 days.

### Polysome profiling

Cells (4 × 10^6^) were treated with 20 µg/ml emetine for 15 min at 37 °C, washed twice with ice-cold PBS/CHX, and scraped. Cells were homogenized by hard shaking with 1.4 mm ceramix spheres (Lysing matrix D MPbio) in FastPrep machine, loaded on 15–50 % sucrose gradient and polysome were fractionated [30]. Fractions were collected with an ISCO (Lincoln, NE) density gradient fractionation system. The settings were as follows: pump speed 0.7 ml/min, fraction time 1 min/fraction, chart speed 150 cm/h and sensitivity of OD254 recorder to 1. The absorbance at 254 nm was measured continuously as a function of gradient depth.

### Cell cycle

Cell pellets (5 × 10^5^ cells) were incubated 1 h on ice before staining overnight at 4 °C with 500 µl of propidium iodide solution (25 µg/ml of propidium iodide, 0.1 % of Triton X100 and 0.1 % of Trisodium citrate dihydrate in water). Analysis was performed by flow cytometry (MACSQuant Analyzer, Miltenyi Biotec).

### Apoptose analysis

Cells (5 × 10^5^) were stained with 125 ng of annexin V-FluoProbes 488 (Interchim) and 7-AAD (1 μg/ml) or Sytox blue (0.1 µg/ml). Cells were incubated for 15 min at RT and analyzed by flow cytometry (MACSQuant Analyzer, Miltenyi Biotec).

### ALDEFLUOR assay

The ALDH enzymatic activity of the cells was measured using the ALDEFLUOR kit (Stem Cell Technologies), according to the manufacturer’s instructions. Briefly, 5 × 10^5^ cells were suspended in ALDEFLUOR assay buffer containing ALDH substrate and incubated for 30 min at 37 °C. As a reference control, cells were stained under identical conditions with the specific ALDH inhibitor diethylaminobenzaldehyde (DEAB). The brightly fluorescent ALDH-expressing cells were detected using a MACSQuant Analyzer, (Miltenyi Biotec). To exclude nonviable cells, Sytox blue was added at a concentration of 0.1 µg/ml.

## Abbreviations

ALDH: Aldehyde dehydrogenase
ATCC: American type culture collection
CRC: Colorectal cancer
FDA: Food and drug administration
P/S: Penicillin–streptomycin
3D: Three-dimensional
TIC: Tumor initiating cells

## References

[1] Jemal A, Bray F, Center MM, Ferlay J, Ward E, Forman D (2011). Global cancer statistics. CA Cancer J Clin.

[2] Haggar FA, Boushey RP (2009). Colorectal cancer epidemiology: incidence, mortality, survival, and risk factors. Clin Colon Rectal Surg.

[3] Schmeichel KL, Bissell MJ (2003). Modeling tissue-specific signaling and organ function in three dimensions. J Cell Sci.

[4] Vermeulen L, Todaro M, de Sousa Mello F, Sprick MR, Kemper K, Perez Alea M, Richel DJ, Stassi G, Medema JP (2008). Single-cell cloning of colon cancer stem cells reveals a multi-lineage differentiation capacity. Proc Natl Acad Sci USA.

[5] Weiswald LB, Richon S, Validire P, Briffod M, Lai-Kuen R, Cordelieres FP, Bertrand F, Dargere D, Massonnet G, Marangoni E (2009). Newly characterised ex vivo colospheres as a three-dimensional colon cancer cell model of tumour aggressiveness. Br J Cancer.

[6] Cohen S, Samadikuchaksaraei A, Polak JM, Bishop AE (2006). Antibiotics reduce the growth rate and differentiation of embryonic stem cell cultures. Tissue Eng.

[7] Keeling KM, Bedwell DM (2002). Clinically relevant aminoglycosides can suppress disease-associated premature stop mutations in the IDUA and P53 cDNAs in a mammalian translation system. J Mol Med (Berl).

[8] Hendrix DV, Ward DA, Barnhill MA (2001). Effects of antibiotics on morphologic characteristics and migration of canine corneal epithelial cells in tissue culture. Am J Vet Res.

[9] El Mouedden M, Laurent G, Mingeot-Leclercq MP, Taper HS, Cumps J, Tulkens PM (2000). Apoptosis in renal proximal tubules of rats treated with low doses of aminoglycosides. Antimicrob Agents Chemother.

[10] Schacht J (1999). Biochemistry and pharmacology of aminoglycoside-induced hearing loss. Acta physiologica, pharmacologica et therapeutica latinoamericana: organo de la Asociacion Latinoamericana de Ciencias Fisiologicas y [de] la Asociacion Latinoamericana de Farmacologia..

[11] Mingeot-Leclercq MP, Tulkens PM (1999). Aminoglycosides: nephrotoxicity. Antimicrob Agents Chemother.

[12] Yang CL, Du XH, Han YX (1995). Renal cortical mitochondria are the source of oxygen free radicals enhanced by gentamicin. Ren Fail.

[13] Whittem T, Schnellmann RG, Ferguson DC (1992). Poly-l-aspartic acid does but triiodothyronine does not protect against gentamicin-induced cytotoxicity in the porcine kidney cell line LLC-PK1. J Pharmacol Exp Ther.

[14] Fukuda Y, Malmborg AS, Aperia A (1991). Gentamicin inhibition of Na+, K(+)-ATPase in rat kidney cells. Acta Physiol Scand.

[15] Laurent G, Kishore BK, Tulkens PM (1990). Aminoglycoside-induced renal phospholipidosis and nephrotoxicity. Biochem Pharmacol.

[16] Pucci B, Kasten M, Giordano A (2000). Cell cycle and apoptosis. Neoplasia.

[17] Kreso A, Dick JE (2014). Evolution of the cancer stem cell model. Cell Stem Cell.

[18] Marcato P, Dean CA, Giacomantonio CA, Lee PW (2011). Aldehyde dehydrogenase: its role as a cancer stem cell marker comes down to the specific isoform. Cell Cycle.

[19] Freedman VH, Shin SI (1974). Cellular tumorigenicity in nude mice: correlation with cell growth in semi-solid medium. Cell.

[20] Malagobadan S, Nagoor NH (2015). Evaluation of microRNAs regulating anoikis pathways and its therapeutic potential. BioMed Res Int.

[21] Bayliss FT, Vinopal RT (1971). Selection of ribosomal mutants by antibiotic suppression in yeast. Science.

[22] Ierusalimskii ND, Shevchenko LA, Grishankova EV (1963). Change of some physiological requirements of yeasts as a result of adaptation to streptomycin. Mikrobiologiia.

[23] Bayliss FT, Ingrahm JL (1974). Mutation in Saccharomyces cerevisiae conferring streptomycin and cold sensitivity by affecting ribosome formation and function. J Bacteriol.

[24] Lamb R, Ozsvari B, Lisanti CL, Tanowitz HB, Howell A, Martinez-Outschoorn UE, Sotgia F, Lisanti MP (2015). Antibiotics that target mitochondria effectively eradicate cancer stem cells, across multiple tumor types: treating cancer like an infectious disease. Oncotarget.

[25] Ennifar E, Paillart JC, Bodlenner A, Walter P, Weibel JM, Aubertin AM, Pale P, Dumas P, Marquet R (2006). Targeting the dimerization initiation site of HIV-1 RNA with aminoglycosides: from crystal to cell. Nucleic Acids Res.

[26] Bose D, Jayaraj G, Suryawanshi H, Agarwala P, Pore SK, Banerjee R, Maiti S (2012). The tuberculosis drug streptomycin as a potential cancer therapeutic: inhibition of miR-21 function by directly targeting its precursor. Angew Chem Int Ed Engl.

[27] Tran TP, Vo DD, Di Giorgio A, Duca M (2015). Ribosome-targeting antibiotics as inhibitors of oncogenic microRNAs biogenesis: old scaffolds for new perspectives in RNA targeting. Bioorg Med Chem.

[28] Pecina-Slaus N, Pecina M (2015). Only one health, and so many omics. Cancer Cell Int.

[29] Sun X, Jiao X, Pestell TG, Fan C, Qin S, Mirabelli E, Ren H, Pestell RG (2014). MicroRNAs and cancer stem cells: the sword and the shield. Oncogene.

[30] David A, Netzer N, Strader MB, Das SR, Chen CY, Gibbs J, Pierre P, Bennink JR, Yewdell JW (2011). RNA binding targets aminoacyl-tRNA synthetases to translating ribosomes. J Biol Chem.

